# Association between development of severe COVID-19 and a polymorphism in the *CIAS1* gene that codes for an inflammasome component

**DOI:** 10.1038/s41598-023-38095-9

**Published:** 2023-07-12

**Authors:** Tania R. Tozetto-Mendoza, Maria Cassia Mendes-Correa, Iara Moreno Linhares, Vanessa de Cássia Raymundi, Heuder Gustavo de oliveira Paião, Erick Matheus Garcia Barbosa, Alessandra Luna-Muschi, Layla Honorato, Giovanna Francisco Correa, Antonio Charlys da Costa, Silvia Figueiredo Costa, Steven S. Witkin

**Affiliations:** 1 Laboratório de Virologia LIM 52, Instituto de Medicina Tropical de São Paulo, Faculdade de Medicina, Universidade de São Paulo, São Paulo, Brazil; 2 Departamento de Moléstias Infecciosas e Parasitárias, Faculdade de Medicina, Universidade de São Paulo, São Paulo, Brazil; 3 Departamento de Ginecologia e Obstetricia, Hospital das Clínicas da Faculdade de Medicina, Universidade de São Paulo, São Paulo, Brazil; 4 Department of Obstetrics and Gynecology, Weill Cornell Medicine, New York, NY USA

**Keywords:** Pathogens, Virology

## Abstract

An elevated pro-inflammatory cytokine response is associated with severe life-threatening symptoms in individuals with Coronavirus Disease-2019 (COVID). The inflammasome is an intracellular structure responsible for generation of interleukin (IL)-1β and IL-18. NALP3, a product of the *CIAS1* gene, is the rate-limiting component for inflammasome activity. We evaluated if a *CIAS1* 42 base pair length polymorphism (*rs74163773*) was associated with severe COVID. DNA from 93 individuals with severe COVID, 38 with mild COVID, and 98 controls were analyzed for this polymorphism*.* The 12 unit repeat allele is associated with the highest inflammasome activity. Five alleles, corresponding to 6, 7, 9, 12 or 13 repeat units, divided into 12 genotypes were identified. The frequency of the 12 unit repeat allele was 45.3% in those with severe disease as opposed to 30.0% in those with mild disease and 26.0% in controls (p < 0.0001, severe vs. controls). In contrast, the 7 unit repeat allele frequency was 30.1% in controls as opposed to 14.0% and 12.5% in those with severe or mild disease, respectively (p ≤ 0.0017). We conclude that individuals positive for the *CIAS1* 12 allele may be at elevated risk for development of severe COVID due to an increased level of induced pro-inflammatory cytokine production.

## Introduction

The current ongoing coronavirus disease-2019 (COVID) pandemic is due to infection with the severe acute respiratory syndrome coronavirus -2 (SARS-CoV-2)^1,2^. The consequences of infection in different individuals range from very mild respiratory manifestations to severe life threatening disease^2^. A characteristic feature of progression to severe COVID is development of a cytokine storm. This is defined as the induction of very high levels of multiple pro-inflammatory cytokines in the circulation that are responsible for immune-related damage to multiple organ systems^3–5^. In addition to age and the presence of concurrent morbidities, the identity of other factors that increase the likelihood of cytokine storm development leading to severe COVID remain incompletely determined^6^.

The mechanism responsible for the formation and release of two biologically active pro-inflammatory cytokines—interleukin (IL)-1β and IL-18—is provided by an intracellular structure called the inflammasome. When microorganisms or their components appear in the cytoplasm, two proteins, NACHT-LRR-PYD-containing protein (NALP) and apoptosis-associated specklike protein, combine to form the inflammasome^7^. This results in NALP activation and its ability to bind to and activate another cytoplasmic protein, caspase-1. Activated caspase-1 is responsible for cleaving the inactive precursors of IL-1β and IL-18 and inducing their release from the cell^8^. IL-1β and IL-18 are both active components of the SARS-CoV-2-induced cytokine storm^6^. IL-1β is produced chiefly by macrophages and epithelial cells, while IL-18 is released from monocytes, macrophages and dendritic cells. They activate the Th17 and Th1 immune cell pathways, respectively, culminating in the release of additional pro-inflammatory mediators^7^.

NALP3 is the member of the NALP family of proteins most associated with infection-induced inflammasome activation. It is a product of the *cold-induced autoinflammatory syndrome 1* (*CIAS1*) gene. Within this gene is a polymorphism, designated *rs74163773*, consisting of a variable number of tandem repeats of a 42 base pair sequence. There can be 6, 7, 9, 12 or 13 repeats of this sequence^9^. The 12 allele is associated with the highest level of NALP3 production and is the rate-limiting step in inflammasome formation^9–11^. Its dominance in individuals has been associated with susceptibility to inflammation-related hypertension^10^ and recurrent vulvovaginal candidiasis^12,13^.

In this communication we tested the hypothesis that carriage of the *CIAS1* 12 allele would increase susceptibility to development of severe COVID in individuals infected with SARS-CoV-2.

## Materials and methods

### Approval

The study was approved by the local ethics committee: Comissão Nacional de Ética em Pesquisa do Ministério da Saúde do Brasil (CONEP), protocol No. CAAE 30419320700000068 and 307019202.00000068, 2020, in accordance with the Declaration of Helsinki. all subjects provided written informed consent. We confirm that all research was performed in accordance with relevant guidelines/regulations.

### Subjects

This study used a convenience sample of participants from two settings: The Corona São Caetano Program^14^ and patients and health care workers from Hospital das Clínicas da Faculdade de Medicina da USP (HCFMUSP). Three groups of individuals were included: 38 patients with mild COVID, 93 patients with severe COVID and 98 controls who were negative for SARS-CoV-2. All samples were collected between May 2020 and September 2021.

### Definitions

COVID status was determined by detection of SARS-CoV-2 on nasal samples or saliva by gene amplification and analysis, as described previously^15^. Health care workers were defined as any worker working within the HCFMUSP, including hospital and auxiliary services. *Mild COVID*: COVID patients who did not required hospitalization at any point in their infection. *Severe COVID*: COVID patients whose infection resulted in hospitalization in the intensive care unit (ICU). *Control group*: Asymptomatic health care workers at HCUSP who tested negative for SARS-CoV-2 by RT-PCR.

### Setting

HCFMUSP is a 2200-bed public teaching hospital designated to receive COVID cases and comprises an emergency department, 300 ICU and 300 ward beds, with 6000 health care workers. Between 30 March and 6 July 2020, 3483 COVID-19 patients were hospitalized.

Health care workers were periodically tested for SARS-CoV-2 by RT-PCR in a surveillance project at HCFMUSP.

The Corona São Caetano Program was a primary care initiative offering COVID care to all residents of São Caetano do Sul, Brazil^14^. Briefly, residents with symptoms consistent with COVID were encouraged to contact the Corona São Caetano platform via a website or by phone. Subjects were excluded from the study if they presented symptoms related to an allergy or bacterial infection and/or who used antibiotics or other medications in the two weeks prior to sample collection. All study subjects had no underlying medical conditions. The respondents were invited to complete an initial screening questionnaire that included information on type, onset and duration of symptoms. Those with symptoms consistent with COVID were contacted by a medical student for further risk assessment. Individuals meeting pre-defined criteria for mild COVID were offered a home visit in which a self-collected nasal-oropharyngeal swabs and a saliva sample were obtained for analysis. Samples from this group were collected from May 5 to May 30, 2020.

### CIAS1 polymorphism analysis

DNA was extracted from peripheral blood or saliva by using automated extractor LOCCUS Biotechnology (Cotia, São Paulo, Brazil), according to the manufacturer's instructions. The DNA samples’ integrity was confirmed by the absence of DNA degradation products in gels. Aliquots were subjected to analysis for the *CIAS1* length polymorphism by gene amplification followed by analysis on 2% agarose gels and visualization by ethidium bromide staining, as described^10,13^. Allele 13 contained 762 base pairs (bp), allele 12 had 720 bp, allele 9 had 594 bp, allele 7 had 520 bp, and allele 6 had 468 bp.

### Statistics

Genotype and allele frequencies were determined by direct counting and then dividing by the number of chromosomes to obtain allele frequency and by the number of women to obtain genotype frequency. Associations between genotype or allele and clinical diagnosis were analyzed by Fisher’s exact test. A *p* value < 0.05 was considered significant.

## Results

### Clinical data

The mean (standard deviation) of subjects’ ages were 47.8 (13.0) years in controls, 41.9 (15.5) years with mild COVID and 56.9 (13.9) with severe COVID. The per cent male subjects was 20.4% in controls, 31.6% in mild COVID and 66.7% in severe COVID.

### Genotype and allele analysis

The five *CIAS1* alleles were combined into 12 different genotypes (Table 1). In individuals with severe COVID (34.4%), the 12,12 genotype was most prevalent. The 13,13 genotype was most prevalent in those with mild COVID (31.6%) as well as in the controls (24.5%). The 12,9 genotype was significantly more prevalent in those with severe COVID (11.8%) as opposed to the controls (1.0%) (p = 0.0021). The prevalence of the 12,9 genotype in those with mild COVID was 5.3%, intermediate between those with severe COVID and no disease. Conversely, the homozygous 7,7 genotype was present in 23.5% of controls as opposed to 8.6% and 5.3% in those with severe or mild COVID, respectively (p ≤ 0.01).

**Table 1:** Genotypes of the *CIAS1* length polymorphism in individuals with COVID-19.

Genotype (alleles)	No. positive (%)			p
	Mild COVID	Severe COVID	Negative	
	N = 38	N = 93	N = 98	
6,6		3 (3.2%)	1 (1.0%)	
7,6			2 (2.0%)	
7,7	2 (5.3%)	8 (8.6%)	23 (23.5%)^a^	0.0134 vs mild COVID 0.0059 vs severe COVID
12,6			2 (2.7%)	
12,7	3 (7.9%)	4 (4.3%)	4 (4.0%)	
9,7	2 (5.3%)	3 (3.2%)	5 (5.1%)	
9,9	7 (18.4%)	10 (10.8%)	10 (10.2%)	
9,13	1 (2.6%)	1 (1.1%)	3 (3.0%)	
12,9	2 (5.3%)	11 (11.8%)	1 (1.0%)^b^	0.0021
12,12	9 (23.7%)	32 (34.4%)	23 (23.5%)	
13,12		4 (4.3%)		
13,13	12 (31.6%)	15 (16.1%)	24 (24.5%)	

Evaluating allele frequencies (Table 2), the 12 allele was present in 45.3% in those with severe COVID, as opposed to 30.0% and 26.0% in those with mild COVID or controls, respectively (p < 0.0001, severe COVID vs. controls). Conversely, the 7 allele frequency was 30.1% in controls.

**Table 2:** Frequency of alleles of the *CIAS1* length polymorphism in individuals with COVID-19.

Allele	No. positive (%)			p
	Mild COVID	Severe COVID	Negative	
	N = 76	N = 186	N = 196	
6	0	8 (2.7%)	4 (2.0%)	
7	9 (12.5%)	23 (14.0%)	59 (30.1%)	0.0017 vs mild COVID < 0.0001 vs severe COVID
9	19 (23.8%)	35 (20.7%)	29 (14.8%)	
12	20 (30.0%)	85 (45.3%)	51 (26.0%)	< 0.0001 vs severe COVID
13	25 (33.8%)	35 (18.0%)	53 (27.0%)	

Taking into consideration just the presence or absence of allele 12 (Table 3), the 12 allele in association with any other allele was present in 22.6% of severe COVID patients as opposed to 13.2% of those with mild COVID and in only 5.1% of controls (p = 0.0005, severe COVID vs. controls). Conversely, the complete absence of the 12 allele was observed in 69.4% of controls, 63.2% of those with mild COVID but in only 43.0% of severe COVID patients (p = 0.0003 vs. controls). In Table 3, “X” indicates the presence of any allele other than allele 12.

**Table 3:** Genotypes of the *CIAS1* length polymorphism in individuals with COVID-19.

Genotype	No. positive (%)			p
	Mild COVID	Severe COVID	Negative	
	N = 38	N = 93	N = 98	
12,12	9 (23.7%)	32 (34.4%)	23 (23.5%)	
12,X	5 (13.2%)	21 (22.6%)	5 (5.1%)	0.0005 vs. severe COVID
X,X	24 (63.2%)	40 (43.0%)	68 (69.4%)	0.0003 vs. severe COVID

## Discussion

Allele 12 of the *CIAS1* length polymorphism was more prevalent in individuals with severe COVID as compared to those with mild COVID or who were uninfected with the SARS-CoV-2 virus. As mentioned above, the concentration of NALP3 is the rate-limiting step in inflammasome formation and individuals positive for the 12 allele produce the highest level of NALP3^9–11^. Thus, carriage of the 12 allele would be expected to result in maximal inflammasome function and the highest production of IL-1β and IL-18. In the absence of allele 12, the inflammasome formed in response to SARS-CoV-2 would have reduced biological activity and suboptimal production and release of active IL-1β and IL-18. It is of interest to point out that disease severity in our subjects were highest in those who were heterozygous rather than homozygous for allele 12. This requires further investigation. It has been reported that IL-1β production by whole blood following ex vivo incubation with a fungal antigen was reduced in individuals possessing the *CIAS1* 7,7 genotype, as compared to those who were 12,12 homozygotes^13^. Another investigation demonstrated that the IL-1β level in peripheral blood was significantly higher in individuals who were allele 12 positive then in those who were negative for this allele^16^. Furthermore, NALP3 has been identified in mucosal epithelial cells that line the oral cavity^17^, enhancing the biological plausibility of inflammasome involvement in mucosal anti-SARS-CoV-2-immune activation at this site.

Two recent studies that also evaluated the association between inflammasome activity and COVID are consistent with our findings. Hadad and co-workers quantitated levels of inflammasome-associated proteins in patients infected with SARS-CoV-2 and reported that measurements of the concentration of these proteins are reliable indicators of the extent of inflammation in these individuals^18^. Rodriques et al. demonstrated that the NALP3 inflammasome is activated by SARS-CoV-2 and the levels of inflammasome components were correlated with disease severity^19^.

Limitations of the current study need to be acknowledged. The total number of subjects analyzed is relatively small, especially those with mild COVID, and our study was not longitudinal. Further investigations on a larger number of individuals positive or negative for COVID are needed to substantiate the present observations, as well as to evaluate long-term outcomes. In addition, although substantiated in prior studies^8,13,16^ we were not able to evaluate our subjects’ sera for the level of IL-1β and IL-18 to directly substantiate the functional consequences of the polymorphism. A further shortcoming is the absence of an analysis of racial differences in our study as a possible explanation for the observed allele variations. It has been demonstrated that the C*IAS1* genotype distribution varies by race^9^. However, we would like to point out that Brazilians are a highly genetically admixed population and determination of race by evaluation of skin color is inaccurate^20,21^. In that context, it is of interest to note that the 13 allele, most frequently detected in African populations and absent in those of European origin^9^, was identified in a high percentage of individuals in our study. Thus, self-reported racial identification in our study population would likely be inaccurate and was not pursued.

## Conclusions

These results implicate genetics as a variable that influences the outcome following a SARS-CoV-2 infection. Prior investigations have implicated additional gene polymorphisms as potential modulators of the consequences of a SARS-CoV-2 infection^22,23^. We would like to stress that redundancy in regulatory mechanisms will almost always limit the consequences of a single genetic variation. Therefore, the need still exists to identify additional gene interactions involved in regulation of the cytokine response to a SARS-CoV-2 infection. When a more comprehensive analysis is obtained of genetic factors associated with development of severe COVID, it would be beneficial to include genetic screening in the initial analysis of SARS-CoV-2-infected individuals.

## Data Availability

All experimental data are available upon request from the corresponding author.
